# Comparison of the Efficacy and Safety of Apixaban and Warfarin in the Prevention of Stroke in Patients With Non-valvular Atrial Fibrillation: A Meta-Analysis

**DOI:** 10.7759/cureus.27838

**Published:** 2022-08-09

**Authors:** Rahat A Memon, Syed Shah Qasim Hamdani, Ali Usama, FNU Aisha, Hayan Kundi, Mohit Mathavan, Malaika Khalid, Areeba Khan

**Affiliations:** 1 Internal Medicine, Abington Memorial Hospital, Abington, USA; 2 Medicine, Foundation University Medical College, Islamabad, PAK; 3 Internal Medicine, Indus Hospital, Lahore, PAK; 4 Medicine, Liaquat University of Medical and Health Sciences, Hyderabad, PAK; 5 Medicine, Fazaia Medical College, Karachi, PAK; 6 Department of Public Health, University of Flordia, Gainesville, USA; 7 Critical Care Medicine, United Medical and Dental College, Karachi, PAK

**Keywords:** atrial fibrillation, meta-analysis, stroke, apixaban, warfarin

## Abstract

Atrial fibrillation is an irregular heart rhythm, and it is one of the most common cardiac arrhythmias. It is associated with a five times increase in the risk of stroke. Anti-coagulants are prescribed routinely to prevent strokes, especially in patients with atrial fibrillation for many years decreasing the risk of stroke among patients with atrial fibrillation. Non-vitamin K oral anticoagulants especially apixaban and rivaroxaban are frequently used and they are considered to be safe and more effective than warfarin. The aim of this meta-analysis is to compare the efficacy and safety of apixaban and warfarin in preventing stroke among patients with non-valvular arterial fibrillation. The current meta-analysis was conducted using the guidelines established by the Preferred Reporting Items for Systematic Reviews and Meta-Analyses (PRISMA). A systematic search was done using databases, including PubMed, EMBASE, and Cochrane Library, with no restrictions on language and year of publication. The current meta-analysis included randomized control trials and non-randomized control trials (prospective and retrospective cohort studies) comparing the efficacy and safety of apixaban and warfarin in preventing stroke in patients with non-valvular atrial fibrillation. The primary efficacy outcome was stroke or systemic embolism while the primary safety outcome was major bleeding events. Overall, nine articles were included in the current meta-analysis with a pooled sample size of 267998 patients with non-valvular atrial fibrillation. The administration of apixaban was associated with a significant decrease in stroke or systemic embolism (RR: 0.77, 95% CI: 0.67-0.90) and major bleeding events (RR=0.63, 95% CI: 0.58-0.68) as compared to warfarin. However, no significant difference was reported in all-cause mortality (RR=0.80, 95% CI: 0.30-2.14) between the two groups. The current meta-analysis concluded that apixaban, compared to warfarin in patients with non-valvular atrial fibrillation showed a reduction in stroke and systemic embolism. Apixaban has also a better safety profile in terms of reduction in overall major bleeding events.

## Introduction and background

Atrial fibrillation is an irregular heart rhythm, and it is one of the most common cardiac arrhythmias. It is associated with a five times increase in the risk of stroke [1]. In the United States, the estimated prevalence of atrial fibrillation was more than 5 million [2]. Anti-coagulants are prescribed routinely to prevent strokes, especially in patients with atrial fibrillation for many years decreasing stroke among patients with atrial fibrillation by 64% as compared to placebo [3]. However, the risk of bleeding is also higher in patients receiving warfarin [4]. Thus, the use of warfarin needs regular international normalized ratio (INR) testing, and it has frequent interactions with multiple medicines and food items [5]. In recent times, a new class of anticoagulants known as non-vitamin K oral anticoagulants has been introduced by scientists [6]. Different clinical trials have shown that non-oral anti-coagulants are equivalent to warfarin in terms of efficacy and safety and thus are routinely prescribed to patients with atrial fibrillation [7-8].

Among the non-vitamin K oral anticoagulants, there are factor Xa inhibitors, including edoxaban, rivaroxaban, and apixaban [9]. A meta-analysis conducted in 2014 to compare warfarin and factor Xa inhibitors found that lower incidence of bleeding and stroke were associated with factor Xa. Among all these medications, apixaban is highly effective in preventing major bleeding events [10]. Apixaban exerts anticoagulant activity by the direct inhibition of the Xa factor that is formed by both extrinsic and intrinsic pathways of coagulation [11]. This prevents the conversion of prothrombin to thrombin, which is needed for the prevention of the formation of fibrin from fibrinogen [11]. Apixaban is approved by the food and drug authority (FDA) in 2011 based on the findings of ARISTOTLE (Apixaban for Reduction in Stroke and Other Thromboembolic Events in Atrial Fibrillation) [12].

As non-vitamin K oral anticoagulants especially apixaban and rivaroxaban are frequently used, new studies are continuously being reported. A meta-analysis including observational studies found a low risk of systemic embolism of stroke and major bleeding with apixaban as compared to warfarin [13]. However, many retrospective observational studies have also been conducted to compare the safety and effectiveness of different non-vitamin K oral anticoagulants. Therefore, we chose to conduct a combined systematic review of experimental and observational studies to further examine and incorporate this new evidence into clinical practice. Our goal was to compare the efficacy and safety of apixaban and warfarin in preventing stroke among patients with non-valvular arterial fibrillation.

## Review

Methodology

The current meta-analysis was conducted using the guidelines established by the Preferred Reporting Items for Systematic Reviews and Meta-Analyses (PRISMA).

Study Selection

A systematic search was done using databases, including PubMed, EMBASE, and Cochrane Library, with no restrictions on language and year of publication. The current meta-analysis included randomized control trials and non-randomized control trials (prospective and retrospective cohort studies) comparing the efficacy and safety of apixaban and warfarin in preventing stroke in patients with non-valvular atrial fibrillation. Studies including participants of 18 years or more with nonvalvular atrial fibrillation using apixaban or warfarin were included. Studies with a follow-up period of fewer than six months after the inception of apixaban or warfarin were excluded from the current meta-analysis. In addition, studies assessing the efficacy of apixaban and warfarin on valvular atrial fibrillation and dialysis patients were also excluded.

A systematic search was performed on July 14, 2022, using the keywords “atrial fibrillation”, “stroke prevention”, “warfarin” and “apixaban”. Keywords were combined using Boolean operators (AND, OR). Keywords were inserted in the medical terms (MeSH) search in PubMed.

Data Collection

Two reviewers independently reviewed the titles and abstracts of each study. Researchers accessed the full text of studies in order to assess whether they fulfilled the eligibility criteria before the process of data extraction. Any disagreement between the two authors was resolved through consensus or discussion with a third investigator if required.

A data collection form was formed on Microsoft Excel (Microsoft Corporation, Redmond, WA) and was shared with other authors. Data related to study type, sample size, intervention, dose, outcomes, inclusion criteria, and follow-up were documented on the data collection form. Outcome data were extracted by two authors independently on a standardized data extraction tool. The data was then transformed to Review Manager (RevMan; [Computer program]. Version 5.4. The Cochrane Collaboration, 2020) and STATA (Stata Statistical Software. College Station, TX: StataCorp LP) for data analysis.

Study Outcomes

The primary efficacy outcome was stroke or systemic embolism while the primary safety outcome was major bleeding, including intracranial bleeding, gastrointestinal bleeding, and bleeding from any other body site. The secondary safety outcome was all-cause mortality.

Assessment of Risk of Bias

Risk is a bias for each article that was assessed by two authors independently. Any disagreement between the two authors was resolved through consensus or discussion with a third investigator if required. For the randomized control trial, the risk of bias was assessed using the Cochrane Risk of Bias tool. To assess the risk of bias in cohort studies, the SIGN methodology was used. Each possible source of bias was categorized as low, moderate, or high.

Statistical Analysis

Statistical analysis was done using the Cochrane Collaboration Review Manager Software (RevMan version 5.4.0) and STATA version 16.0 (Version 16, StataCorp, College Station, Texas). The Mantel-Haenszel (M-H) random-effects meta-analysis model was used and forest plots were utilized to present treatment effect as risk ratio (RR) and 95% confidence interval (CI). A p-value ≤ 0.05 was considered statistically significant. For quantitative measurement of inconsistency, I2 statistics were used. Cochran's Q test was used for statistical testing of heterogeneity. A p-value less than 0.1 will be considered significant for heterogeneity. To assess publication bias, Egger’s regression test was used and a p-value ≤ 0.05 was considered significant for publication bias.

Results

**Figure 1:**
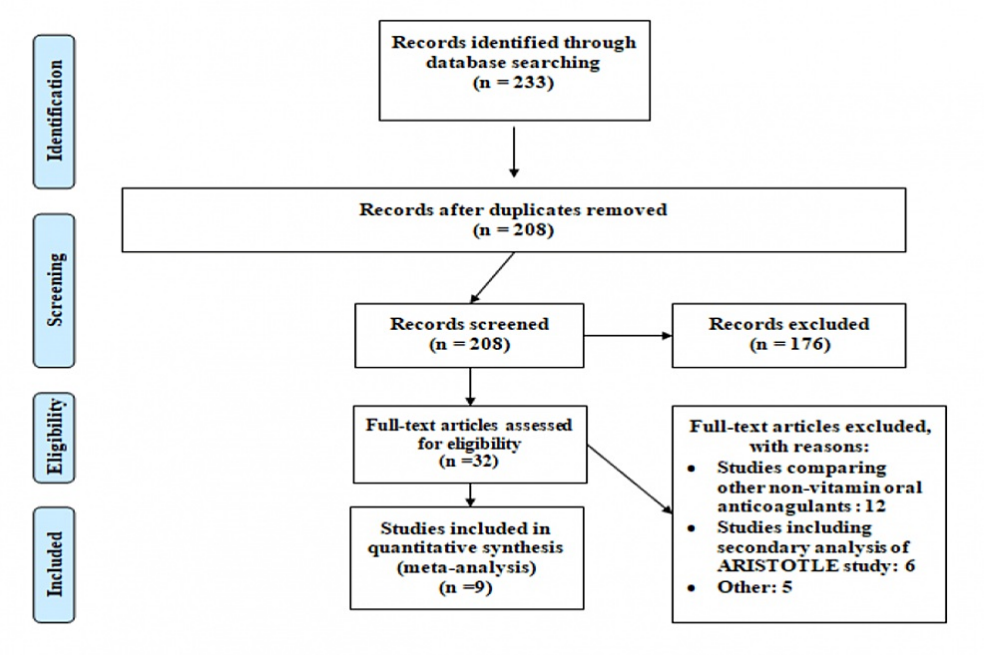
PRISMA flow chart of selection of studies

Through a systematic database search, 233 studies were identified. After removing duplicates, the titles and abstracts of 208 articles were screened. A PRISMA flow diagram representing the selection of studies is shown in Figure 1. Overall, the full texts of 32 articles were retrieved for assessment of eligibility. Overall, nine articles were included in the current meta-analysis with a pooled sample size of 267998 patients with atrial fibrillation (96,631 in the apixaban group and 171,367 in the warfarin group). Characteristics of the eligible studies are presented in Table 1.

**Table 1:** Study characteristics AF: atrial fibrillation; OAC: oral anticoagulants; NVAF: non-valvular atrial fibrillation Reduced dose: 2.5 mg Standard dose: 5.0 mg

Author	Year	Study Type	Groups	Dose	Sample size	Follow-up	Inclusion Criteria	Reduced dose of apixaban
Fu et al [14]	2021	Retrospective cohort	Apixaban	Reduced or standard dose	1625	12 Months	Adult patients with non-valvular AF.	915 (56.31%)
			Warfarin	Reduced or standard dose	1625			
Granger et al [12]	2011	Randomized trial	Apixaban	Reduced or standard dose	9120	24 Months	Eligible patients had atrial fibrillation or flutter at enrollment or two or more episodes of atrial fibrillation or flutter and age of at least 75 years	428 (4.7%)
			Warfarin	Standard dose	9081			
Gupta et al [15]	2018	Retrospective cohort	Apixaban	Reduced or standard dose	7607	6 Months	Adult patients with non-valvular AF.	2428 (21.7%)
			Warfarin	Standard dose	7607			
Larsen et al [16]	2016	Observational cohort	Apixaban	Standard dose	6349	30 Months	Patients with atrial fibrillation who had not previously taken an oral anticoagulant.	NA
			Warfarin	Standard dose	35436			
Li et al [17]	2017	Observational cohort	Apixaban	Reduced or standard dose	38470	12 Months	Patients age >=18 years with atrial fibrillation	6568 (17.1%)
			Warfarin	Standard dose	38470			
Kohsaka et al [18]	2018	Retrospective cohort	Apixaban	Reduced or standard dose	11972	6 Months	Patient of age of 18 years or more and diagnosis of atrial fibrillation and prescribed one of the two study drugs (apixaban or warfarin) after diagnosis of atrial fibrillation	7,251 (60.6%)
			Warfarin	Standard dose	11972			
Nielsen et al [19]	2017	Observational cohort	Apixaban	Reduced dose	4400	30 Months	Patients with atrial fibrillation who had not previously taken an oral anticoagulant.	4400 (100%)
			Warfarin	Standard dose	38893			
Staerk et al [20]	2017	Observational Cohort	Apixaban	Standard dose	6899	24 Months	AF patients with no previous OAC treatment before the study period were included on the day	NA
			Warfarin	Standard dose	18094			
Wanat et al [21]	2019	Retrospective cohort	Apixaban	Standard dose	10189	12 Months	Patients were included if they were aged 18 years or older with a diagnosis of NVAF and receiving either warfarin or apixaban	NA
			Warfarin	Standard dose	10189			

Among all the included studies, only one article was a randomized control trial [12] while other studies were either observational cohorts [16-18,20] or retrospective cohorts [14-15,19,21]. One study was published in 2011 [12] while other studies were published between 2016 and 2021 [14-21]. One study included patients in which only a reduced dose (2.5 mg) of apixaban was given [19] while the majority of studies included patients in which patients taking both reduced (2.5 mg) and standard (5 mg) of apixaban [12,14-15,17-18].

Risk of Bias Evaluation

Table 2 shows the risk of bias evaluation of all nine studies. Five of the included studies have low overall bias while four studies reported moderate overall bias. No significant publication bias was found in the primary efficacy endpoint and secondary efficacy endpoint, for comparison between warfarin and apixaban as a p-value of the Egger regression test was >0.05.

**Table 2:** Risk of bias assessment

Study Id	Selection bias	Attrition bias	Performance bias	Detection bias	Reporting bias	Overall bias
Fu et al, 2012 [14]	High	Low	Low	Low	Low	Moderate
Granger et al, 2011 [12]	Low	Low	Low	Low	Low	Low
Gupta et al, 2018 [15]	Low	Low	Low	Low	Low	Low
Larsen et al, 2016 [16]	High	Low	Low	Low	Low	Moderate
Li et al, 2017 [17]	Low	Low	Low	Low	Low	Low
Kohsaka et al, 2018 [18]	Low	Low	Moderate	Low	Low	Moderate
Nielsen et al, 2017 [19]	High	Low	Moderate	Low	Low	Moderate
Staerk et al, 2017 [20]	Low	Low	Low	Low	Low	Low
Wanat et al, 2019 [21]	Low	Low	Low	Low	Low	Low

Efficacy Outcome

**Figure 2:**
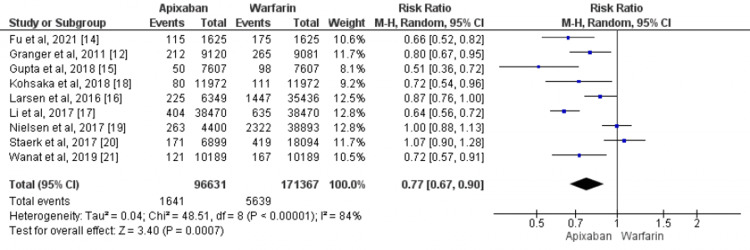
Comparison of the effect of apixaban and warfarin on the risk of stroke and systemic embolism Source: References [12,14-21]

All the included articles reported the primary efficacy outcome [12,14-21], including 267998 patients with available data in terms of stroke or systemic embolism. The risk of stroke or systemic embolism is 23% lower in patients receiving apixaban as compared to patients receiving warfarin (RR: 0.77, 95% CI: 0.67-0.90). Heterogeneity was significant among the studies (I2 = 84%, p-value=0.001) as shown in Figure 2.

Safety Outcome

**Figure 3:**
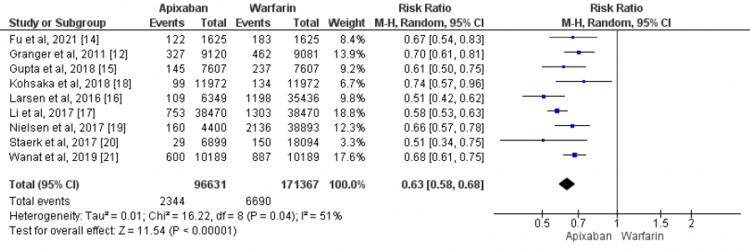
Comparison of the effect of apixaban and warfarin on the risk of major bleeding events Sources: [12,14-21]

All the included studies reported primary safety outcomes, i.e. major bleeding, including 267998 patients with atrial fibrillation [12,14-21]. The administration of apixaban was associated with a significant reduction in major bleeding events compared with warfarin (RR=0.63, 95% CI: 0.58-0.68). Heterogeneity was significant among the studies (I2 = 51%, p-value=0.040) as shown in Figure 3.

All-Cause Mortality

**Figure 4:**
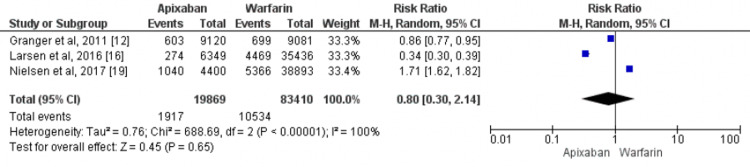
Comparison of the effect of apixaban and warfarin on all-cause mortality Sources: [12,16,19]

Three studies compared all-cause mortality in patients with atrial fibrillation receiving apixaban and warfarin [12,16,19]. No significant difference was found in the incidence of all-cause mortality between patients who received apixaban and patients who received warfarin (RR=0.80, 95% CI: 0.30-2.14) as shown in Figure 4.

Heterogeneity

A high level of statistical heterogeneity was noted in the current meta-analysis for all the outcomes assessed in the study. One of the major reasons for high heterogeneity among the study results is the type of study design. Only one RCT was present [12] while four studies were retrospective cohorts [14-15,19,21] and four studies were observational cohorts [16-18,20]. Second, two studies included only a standard dose [16,20-21] while the majority of studies used both a standard dose and a reduced dose of apixaban [12,14-15,17-18]. It might be another cause of high heterogeneity among the study results.

Sensitivity Analysis

Table 2 shows the results of sensitivity analysis of the risk of stroke or systemic embolism and major bleeding events. We performed a sensitivity analysis of the risk of stroke or systemic embolism by excluding the randomized control trial and retrospective studies and the results were inconsistent as compared to the overall analysis (RR=0.87, 95% CI: 0.69-1.10). When including only retrospective cohort studies, heterogeneity was reduced to 6%, and results found that apixaban is better in reducing strokes or systemic embolism as compared to warfarin (RR=0.66, 95% CI: 0.58-0.76). On the other hand, when it comes to major bleeding events, results reported in sensitivity analysis are consistent with the overall analysis as shown in Table 2.

**Table 3:** Results of sensitivity analysis Significant at p-value<0.05 RR: risk ratio; CI: confidence interval

Outcome	Included Studies	RR (95% CI)	I2
Stroke or systemic embolism	Randomized trial	0.80 (0.67-0.95)*	-
	Observational cohort	0.87 (0.69-1.10)	89%
	Retrospective cohort	0.66 (0.58-0.76)*	6%
Major bleeding events	Randomized trial	0.70 (0.61-0.81)*	-
	Observational cohort	0.58 (0.52-0.64)*	39%
	Retrospective cohort	0.67 (0.62-0.73)*	0%

Discussion

The current meta-analysis was conducted to compare the efficacy of apixaban and warfarin in the prevention of stroke among patients with atrial fibrillation. Overall nine studies were included in the current meta-analysis, including a pooled sample size of 267998 patients with atrial fibrillation. The study found that patients who were taking apixaban had less stroke or systemic embolism and fewer major bleeding events as compared to patients taking warfarin, and the results were statistically significant.

Previous meta-analyses conducted by Proietti et al. [13] and Siddiqui et al. [10] favored apixaban over warfarin in terms of safety. However, no significant differences were reported in terms of the prevention of stroke between apixaban and warfarin. However, a meta-analysis conducted by Proietti et al. did not include the ARISTOTLE trial in which apixaban had a significant impact on the reduction of stroke or systemic embolism along with events of major bleeding compared to warfarin [13]. On the other hand, Siddiqui et al. did not include retrospective studies that found similar results [10]. Compared to a meta-analysis conducted in the past, we have included retrospective studies in the current meta-analysis. However, outcomes have remained the same. Standard-dose apixaban is discovered to have comparable efficacy but improved safety when compared to warfarin, supporting earlier meta-analyses. There is still controversy around the efficacy of reduced-dose apixaban, and more prospective studies are required.

The dose of apixaban is a significant influencing factor in its safety and efficacy. Even though the apixaban label shows a dose of 5 mg twice daily for patients with non-valvular atrial fibrillation, patients who meet any of the two following criteria are recommended to take a dose of 2.5 mg twice daily: age 80 years or more, a body weight of fewer than 60 years, and serum creatinine of 1.5 mg/dl or more [22]. The secondary analysis of the ARISTOTLE trial showed that no significant difference was there in terms of prevention of stroke between warfarin and reduced-dose apixaban, but on comparison between reduced dose and standard dose apixaban, the risk of stroke or systemic embolism was 23% lower in standard-dose apixaban [23]. Our meta-analysis had four studies that compared reduced-dose apixaban with warfarin. As discussed in individual articles included in the current meta-analysis, reduced dose apixaban is prescribed in individuals with old age or patients with at least two comorbidities. Gupta et al. conducted a study also found that no significant difference was there between in incidence of stroke between warfarin and reduced dose apixaban while standard dose apixaban is more effective in preventing stroke or systemic embolism than warfarin [15].

From our analysis, the superiority of apixaban over warfarin in reducing the rate of stroke and systemic embolism is evident. To date, only one randomized control trial has been conducted on this topic, which also shows the clinical benefit of apixaban over warfarin in atrial fibrillation patients [12]. However, several new prospective cohort studies and retrospective studies have been conducted. Besides this, the superiority of several other novel oral anticoagulants over warfarin in decreasing stroke and systemic embolism is evident in different studies [24-25]. Due to this, nowadays, novel oral anticoagulants are being utilized in practice settings and are being recommended by several professional organizations [26].

The current meta-analysis has certain limitations. First, there is a lack of prospective studies and randomized trials. Second, heterogeneity was high in the included studies as shown by the value of I2. Third, only three studies were included in the meta-analysis that compared the impact of apixaban and warfarin on all-cause mortality. In the future, more prospective studies need to be conducted to study the effect of apixaban and warfarin on different subgroups, including patients with valvular atrial fibrillation and various valve diseases.

## Conclusions

The current meta-analysis demonstrated that apixaban, compared to warfarin, in patients with atrial fibrillation showed a reduction in stroke and systemic embolism. Apixaban has also a better safety profile in terms of reduction in overall major bleeding events. However, the current study did not report any significant difference between all-cause mortality between apixaban and warfarin. The current meta-analysis included randomized trials, prospective cohorts, and retrospective studies on this topic. This reviewer reinforces apixaban's superiority in comparison to warfarin in patients with non-valvular atrial fibrillation.
